# Correction: Tian et al. Diaporine Potentiates the Anticancer Effects of Oxaliplatin and Doxorubicin on Liver Cancer Cells. *J. Pers. Med.* 2022, *12*, 1318

**DOI:** 10.3390/jpm16020101

**Published:** 2026-02-09

**Authors:** Shiliu Tian, Rui Su, Ke Wu, Xuhan Zhou, Jaydutt V. Vadgama, Yong Wu

**Affiliations:** 1 Key Laboratory of Exercise and Health Sciences of Ministry of Education, Shanghai University of Sport, Shanghai 200438, China; tianshiliu@sus.edu.cn; 2 Fujian Sports Vocational Education and Technical College, Fuzhou 350001, China; 3 College of Engineering, University of California, Berkeley, CA 94720, USA; rui999@berkeley.edu; 4 David Geffen UCLA School of Medicine and UCLA Jonsson Comprehensive Cancer Center, Division of Cancer Research and Training, Department of Internal Medicine, Charles Drew University of Medicine and Science, Los Angeles, CA 90095, USA; kewu@cdrewu.edu (K.W.); jayvadgama@cdrewu.edu (J.V.V.); 5 Fulgent Life Inc., Irvine, CA 92620, USA; jameszhou201801@gmail.com

## Incorrect Citation

In the original publication [1], References 1–3 cited in the Introduction were not the most appropriate sources to support the general epidemiological statement that cancer is one of the main causes of death and morbidity worldwide. The originally cited references primarily addressed specific cancer types or therapeutic strategies and did not provide comprehensive global cancer incidence and mortality statistics.

To correct this issue, References 1–3 have been replaced with authoritative global cancer statistics reports published prior to the submission and acceptance of the original article. The corrected references are as below:

1. Bray, F.; Ferlay, J.; Soerjomataram, I.; Siegel, R.L.; Torre, L.A.; Jemal, A. Global cancer statistics 2018: GLOBOCAN estimates of incidence and mortality worldwide for 36 cancers in 185 countries. *CA Cancer J. Clin.* **2018**, *68*, 394–424.
2. Sung, H.; Ferlay, J.; Siegel, R.L.; Laversanne, M.; Soerjomataram, I.; Jemal, A.; Bray, F. Global cancer statistics 2020: GLOBOCAN estimates of incidence and mortality worldwide for 36 cancers in 185 countries. *CA Cancer J. Clin.* **2021**, *71*, 209–249.
3. Ferlay, J.; Colombet, M.; Soerjomataram, I.; Parkin, D.M.; Piñeros, M.; Znaor, A.; Bray, F. Cancer statistics for the year 2020: An overview. *Int. J. Cancer* **2021**, *149*, 778–789.

The authors state that the scientific conclusions are unaffected. This correction was approved by the Academic Editor. The original publication has also been updated.
